# Characterization of a Novel Porcine *CSN2* Polymorphism and Its Distribution in Five European Breeds

**DOI:** 10.3390/ani9070419

**Published:** 2019-07-04

**Authors:** Mihai Șuteu, Augustin Vlaic, Stelian Vasile Dărăban

**Affiliations:** Faculty of Animal Husbandry and Biotechnologies, University of Agricultural Sciences and Veterinary Medicine, 3-5 Calea Mănăștur Street, 400372 Cluj-Napoca, Romania

**Keywords:** pig, *Sus scrofa*, *CSN2*, polymorphism

## Abstract

**Simple Summary:**

Polymorphisms in genes encoding major milk proteins have been intensely studied, and are used, worldwide, in marker-assisted selection in dairy species: *αS1-casein* in goats—associated with milk protein content and flavor; *κ-casein* and *β-lactoglobulin* in cattle—associated with milk quantity and quality; bovine *β-casein*—associated with human health, etc. This aspect has scarcely been investigated in pigs. Using an electrophoretic technique, we previously identified a novel porcine β-casein (encoded by *CSN2*) polymorphism. Here, we fully characterize it at protein and DNA level, propose a genotyping protocol, and investigate its distribution in five European porcine breeds. In brief, a G/A point mutation in position 647 of the porcine *CSN2* cDNA leads to an arginine/glutamine substitution in position 201 of the protein. This mutation can be typed via a *StyI* PCR-RFLP assay. The frequency of the G allele was 0.965 in the investigated Landrace population (number of individuals genotyped *n* = 67), one in the Pietrain (*n* = 40), 0.705 in the Large White (*n* = 36), 0.885 in the Bazna (*n* = 13), and 0.555 in the Mangalita population (*n* = 11). Considering that milk protein content still varies widely within (and between) porcine breeds, this and/or other similar polymorphisms may have implications for the dynamics of piglet growth during suckling.

**Abstract:**

Here, we describe a novel porcine *β-casein* (*CNS2*) polymorphism, initially identified using the isoelectric focusing (IEF) technique, and provide its distribution in five European breeds. Porcine *CSN2* cDNA samples, from sows identified using IEF as carriers of polymorphic variants, were sequenced, and based on the sequence alignments, a genotyping assay was developed. The distribution of the polymorphism was investigated by genotyping 167 sows. Population genetic indexes were computed using POPGENE32 version 1.32. Sequence alignments revealed that the mutation which caused the different β-casein IEF migration profiles was c.647G>A, a substitution located in exon 7, which modifies the amino acid from position 201 of the mature protein from arginine to glutamine. The frequency of the G allele was 0.965 in the investigated Landrace population (number of individuals genotyped *n* = 67), one in the Pietrain population (*n* = 40), 0.705 in the Large White population (*n* = 36), 0.885 in the Bazna population (*n* = 13), and 0.555 in the Mangalita population (*n* = 11). For all breeds, except Pietrain (monomorphic), the genotype distribution was in accordance with the Hardy–Weinberg equilibrium. Given that β-casein is the most important protein in sows’ milk, a polymorphism like the one described here may prove interesting for marker-assisted selection.

## 1. Introduction

Caseins are the most abundant proteins in the milk of most mammalian species (>60% in swine), and consist primarily of a group of heterogeneous phosphoproteins [1]. Of the four caseins, β-casein is the most abundant, with an average content of 37% in cattle milk and more than two-fold content compared to αS1-casein in porcine milk [1,2,3].

Polymorphism of milk proteins in dairy species has been intensely studied, at both protein and DNA level [4]. In the case of pigs, this aspect was scarcely investigated and most of the work was conducted using protein-based techniques, reviewed by Gallagher et al. [3]. According to the same authors, it is difficult to interpret the early reports on porcine milk proteins as no official protein nomenclature system was used, and therefore, some of the proteins may have been incorrectly named [3].

The first sequence of the cDNA of the gene encoding β-casein in pigs (gene referred to hereafter as *CSN2*) reported a cDNA length of 1100 bp, excluding the poly(A) tail, encoding a mature protein of 217 amino acids [5]. 

The first *CSN2* polymorphism, identified at DNA level, was a *SacI* type polymorphism, the same authors mapping *CSN2* to chromosome 8 [6]. More recent studies, conducted at DNA level, revealed that porcine *CSN2* is highly polymorphic: A new allele was characterized [7], four SNPs were identified [8], an alternative splicing phenomenon was documented [9,10], and, most notably, a SNP in the gene’s TATA box, leading to decreased promoter activity, was described [11]. 

In a previous study [12], using isoelectric focusing (IEF), we identified a new porcine β-casein polymorphism and reported two polymorphic variants. A comparison with previous studies that reported porcine β-casein polymorphisms using protein-based assays, as reviewed by Gallagher et al. [3], was not possible in light of the different techniques used. The present paper fully characterizes the novel polymorphism and highlights its distribution in five European porcine breeds. The breeds were selected to cover both highly improved and indigenous unimproved breeds.

## 2. Materials and Methods 

### 2.1. SNP Characterization 

The Faculty of Animal Husbandry and Biotechnologies, University of Agricultural Sciences and Veterinary Medicine, Cluj-Napoca, Romania approved the study design and procedures.

Total RNA samples were extracted, from milk somatic cells, from sows identified by IEF as carriers of polymorphic *CSN2* variants. Three of the milk samples, obtained by hand milking, from the initial study [12] were used. From these, porcine *CSN2* cDNA samples were obtained and sequenced. All techniques (IEF, RNA isolation, reverse-transcription, and cDNA sequencing) are described in detail in our previous papers on the subject [7,9,12]. Sequence alignments and theoretical translations (DNA to protein) were performed to characterize the polymorphism.

### 2.2. Genotyping

Sequence alignments revealed that the mutation causing the different IEF migration profiles was c.647G>A, a substitution located in exon 7 of the porcine *CSN2* gene. As such, the following set of primers was designed to amplify a DNA fragment harboring the SNP:

F 5’-3’: CTG AAG ACC AAA GTA AGT AGC

R 5’-3’: TAT TCC AAG CCA CAT GAG AT

A standard PCR consisting of 35 cycles (94 °C/1 min, 60 °C/1 min, 72 °C/1 min) was used to produce a 1216 bp fragment containing the mutation site.

The construction of theoretical restriction maps [13] showed that the *StyI* endonuclease would allow us to differentiate between these two allelic variants. It cuts the 1216 bp amplicon in three fragments in the case of G allele (1113, 85 and 15 bp), while the digestion of A allele yields four fragments (679, 434, 85, and 18 bp). Heterozygous individuals have all five fragments (1113, 679, 434, 85 and 18 bp). Restriction using *StyI* (Fermentas FastDigest) was performed in 200 μL tubes in the thermocycler, to ensure complete digestion, following the manufacturer’s recommendations. 

Using the PCR-RFLP protocol described above, the c.647G>A porcine *CSN2* polymorphism was investigated in 167 sows belonging to five European breeds. The samples were collected from 2 farms in Belgium (Landrace, *n* = 67; Pietrain, *n* = 40; Large White *n* = 36) and 1 farm in Romania (Bazna, *n* = 13; Mangalita, red variety, *n* = 11).

### 2.3. Statistical Analysis

Allelic frequencies and chi-square values were calculated using the standard formulae. Population genetic indexes (gene homozygosity, gene heterozygosity, effective number of alleles, and fixation index) were computed using POPGENE32 version1.32 [14], as done by Selvaggi et al. [15].

## 3. Results

### 3.1. SNP Characterization

**Figure 1:**
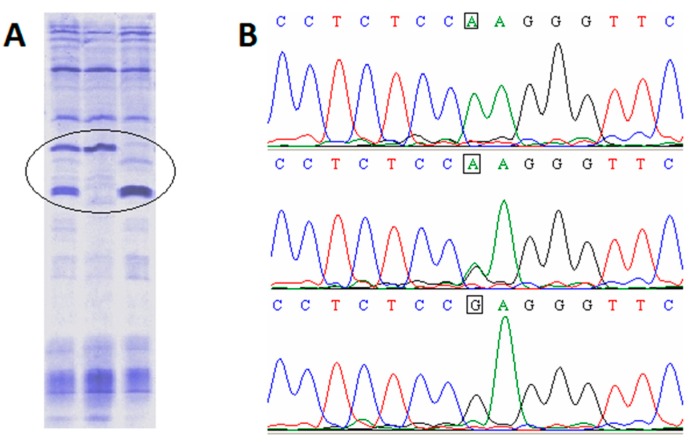
Porcine *CSN2* c.647G>A polymorphism: (**A**) The isoelectric focusing (IEF) migrating profiles of the two polymorphic protein variants (lane 1—AG; lane 2—AA; lane 3—GG); (**B**) *CSN2* cDNA sequencing chromatograms from 3 individuals carrying different genetic variants of the gene—the mutational event is marked with a square.

Sequencing the *CSN2* cDNA from three reference samples (Figure 1A) confirmed at DNA level the polymorphism previously identified at protein level [12]. Figure 1B shows the fragment of the sequencing chromatograms where the substitution that caused the occurrence of the two protein variants is located (position 647 of the cDNA).

The c.647G>A substitution modifies the amino acid from position 201 of the mature protein; the CGA codon encodes arginine (R), CAA codon encodes glutamine (Q). The sequencing results are in accordance with the IEF results. The R in position 201 gives the mature protein an isoelectric point (pI) of 5.99—a fact pointed out at IEF level by a more cathodic migration profile. If Q is present, the isoelectric point of the protein becomes pI=5.77—conferring the protein a more anodic migration profile, compared to the A variant. Of course, the IEF migration profile of the porcine β-casein in the case of heterozygous individuals is characterized by the presence of both bands. 

The alignment of the amino acid sequences of the two polymorphic porcine β-casein variants, based on theoretical translation (not shown) indicates that from a total of 217 amino acids constituting the mature protein, 216 are identical, the only difference being in the 201st position.

In most known DNA sequences, adenine was reported in this position (GenBank NM_214434, X54974, EU025876, EU213063), while only two sequences deposited in GenBank reported the presence of guanine (EU242520, GU827390). 

### 3.2. Genotyping

**Figure 2:**
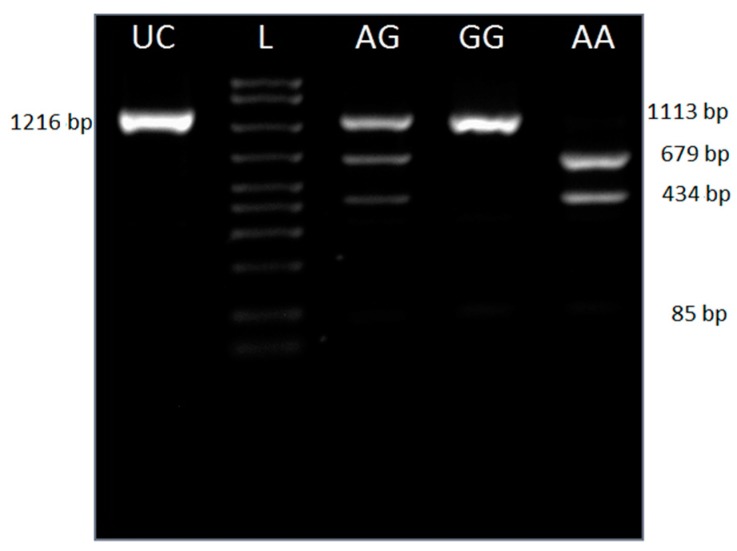
Agarose gel electrophoretic profiles belonging to the 3 porcine *CSN2* c.647G>A genotypes, following the restriction of amplification products with the *StyI* endonuclease. Lane 1—unrestricted product; lane 2—AmpliSize Molecular Ruler 50–2000 bp Ladder (BioRad); lane 3—AG; lane 4—GG; lane 5AA.

The proposed *StyI* PCR-PFLP is able to differentiate between the two allelic variants, as shown in Figure 2 (the 18 bp fragment, due to its small size, cannot be visualized).

Genotyping results obtained with the proposed protocol are provided in Table 1. Gene and genotype frequencies show that, in all investigated breeds, the *CSN2* c.647G variant is predominant. The highest frequency of c.647A is encountered in the case of the Bazna breed (0.445). Concerning this SNP, the locus is monomorphic in the case of the Pietrain breed (Table 1).

### 3.3. Statistical Analysis

The computed population indexes are given in Table 2. Except for the Pietrain breed, where the genetic indexes were not computed (monomorphic locus), for all the investigated populations, the genotype distribution is in accordance with the Hardy–Weinberg equilibrium (χ^2^_tab_ = 3.48, d. f. = 1).

Excess of heterozygosity, as compared to the equilibrium expectations, was observed for all breeds (i.e., negative F_IS_ values); this was very low in the case of the Landrace breed.

## 4. Discussion

In this paper, we fully characterized for the first time porcine *CNS2* c.647G>A polymorphism. This mutation, although never described, was previously inferred by sequence alignments [7]. From a nomenclature standpoint, this mutation does not allow allelic discrimination based on the previously proposed naming system, i.e., other mutations differentiate the known *CSN2* sequences, and haplotypes would need to be considered [16]. 

Knowledge regarding the distribution of the polymorphism and the population indexes is important both for the indigenous unimproved breeds (Bazna and Mangalita—detailed breed descriptions provided by Ciobanu and colleagues [17]), and also for the cosmopolitan breeds included in the study (Landrace, Large White, and Pietrain). 

The two indigenous breeds are in danger of extinction [18], so knowing the allelic frequencies and population indexes is important from a biodiversity point of view. In this respect, the excess of heterozygotes seen in our study (compared to the equilibrium expectations) is in line with the findings of Zăhan and colleagues [19], who used microsatellite markers to genetically characterize the Romanian Mangalita population.

For the cosmopolitan breeds, well established in commercial hybrid production schemes, this polymorphism could prove interesting from a selection point of view. Polymorphisms within the genes encoding major milk proteins are known to be associated with milk quality (e.g., protein content), and are implemented in marker-assisted selection in dairy species [4]. It is well known that the largest input cost in the pig industry is feed. As increased weaning weight was reported to significantly reduce the age at slaughter [20,21], a higher maternal milk protein content, which could be expected to increase the weaning weight, would translate into immediate financial benefits. Despite indirect selection, by selecting for sow nursing capacity, or litter weight at weaning, there is still a high variability in sow milk protein content [22]. Accordingly, given that β-casein is the most abundant protein in sow milk, a *CSN2* polymorphism may prove interesting for marker-assisted selection. Attempts to correlate porcine β-casein polymorphisms with piglet growth dynamics were already initiated [23], but the methods used (i.e., horizontal starch gel electrophoresis) do not allow us to link the reported findings to this novel polymorphism.

Tributary to its fundamental approach, one major limitation of the present work is the lack of association studies between this polymorphism and milk protein content and/or piglet growth dynamics during suckling, and such aspects should be evaluated in future research.

It is well known that only a trait showing variability offers the possibility for selection and sow milk protein content is such a trait. As the gene encoding one of its major constituents, namely *CNS2*, exhibits such a high degree of polymorphism, we reiterate the hypothesis that *CSN2* polymorphisms, via impact on the gene’s expression, are associated with sow milk protein content.

## Figures and Tables

**Table 1:** *CSN2* c.647G>A polymorphism: Genotype and gene frequencies in different breeds.

Breed	N	Number of Sows			Genotype Frequency			Gene Frequency	
		AA	AG	GG	P_AA_	H_AG_	Q_GG_	p_A_	q_G_
Landrace	67	0	3	64	0	0.07	0.93	0.035	0.965
Pietrain	40	0	0	40	0	0	1	0	1
Large White	36	2	17	17	0.06	0.47	0.47	0.295	0.705
Mangalita	13	0	3	10	0	0.23	0.77	0.115	0.885
Bazna	11	1	8	2	0.09	0.73	0.18	0.445	0.545

*CSN2*, the gene encoding β-casein; N, number of genotyped individuals.

**Table 2:** Population genetic indexes for *CSN2* c.647G>A, within the investigated populations.

Breed	Gene Homozygosity (Ho)	Gene Heterozygosity (He)	Effective Number of Alleles (Ne)	Fixation Index (F_IS_)	Hardy-Weinberg Equilibrium (χ2)
Landrace	0.9552	0.0448	1.0458	−0.0229	0.0232
Large White	0.5278	0.4722	1.7041	−0.1429	0.6044
Mangalita	0.2727	0.7273	1.9836	−0.4667	1.9394
Bazna	0.7692	0.2308	1.2565	−0.1304	0.1423

*CSN2*, the gene encoging β-casein.
